# ZhenQi FuZheng formula‐mediated improvement of hematopoietic function in cyclophosphamide‐treated mice via the upregulation of macrophage colony‐stimulating factor concentrations

**DOI:** 10.1002/ctm2.256

**Published:** 2020-12-16

**Authors:** Dongjie Li, Qiubo Chu, Shimiao Wang, Lanzhou Li, Bo Dou, Jiawei He, Yaping Tian, Di Wang

**Affiliations:** ^1^ Department of Otorhinolaryngology Head and Neck Surgery The First Hospital of Jilin University Jilin P.R. China; ^2^ School of Life Sciences Jilin University Jilin P.R. China; ^3^ Department of Dermatology and Venerology The First Hospital of Jilin University Jilin P.R. China

Dear Editor,

Owing to chronic radiotherapy and chemotherapy, cancer patients may suffer from immunosuppression, hematopoietic inhibition, and myelosuppression.^1^ In this study, we first evaluated the effect of ZhenQi FuZheng formula (ZQFZ) on hematopoiesis in cells of mice with immunosuppression and hematopoietic dysfunction.

The stability of the six effective constituents of ZQFZ according to the Chinese Pharmacopoeia (Version 2015) was first analyzed by high‐performance liquid chromatography (HPLC) (Figure S1).

Hematopoietic stem cells (HSCs) have been reported to maintain the hematopoietic function via the dynamic balance between their proliferation, the differentiation of red blood cells, and the formation of blood cells; thus, the agents that protect and/or improve the self‐renewal of HSCs may be potential candidates for developing anti‐myelosuppression drugs.^1^ ZQFZ strongly increased the proliferation of K562 cells (human myelogenous leukemia) and CHRF cells (human megakaryoblastic leukemia) (Figure 1A) without influencing their apoptosis (Figure 1C and D), motivated erythrocyte transformation (Figure 1B), and regulated the expressions of proteins, including P‐RSK1‐p90, c‐Myc, and ETS transcription factor ELK1, which are associated with erythroid differentiation in the hematopoietic system (Figure 1E and F).

**FIGURE 1:**
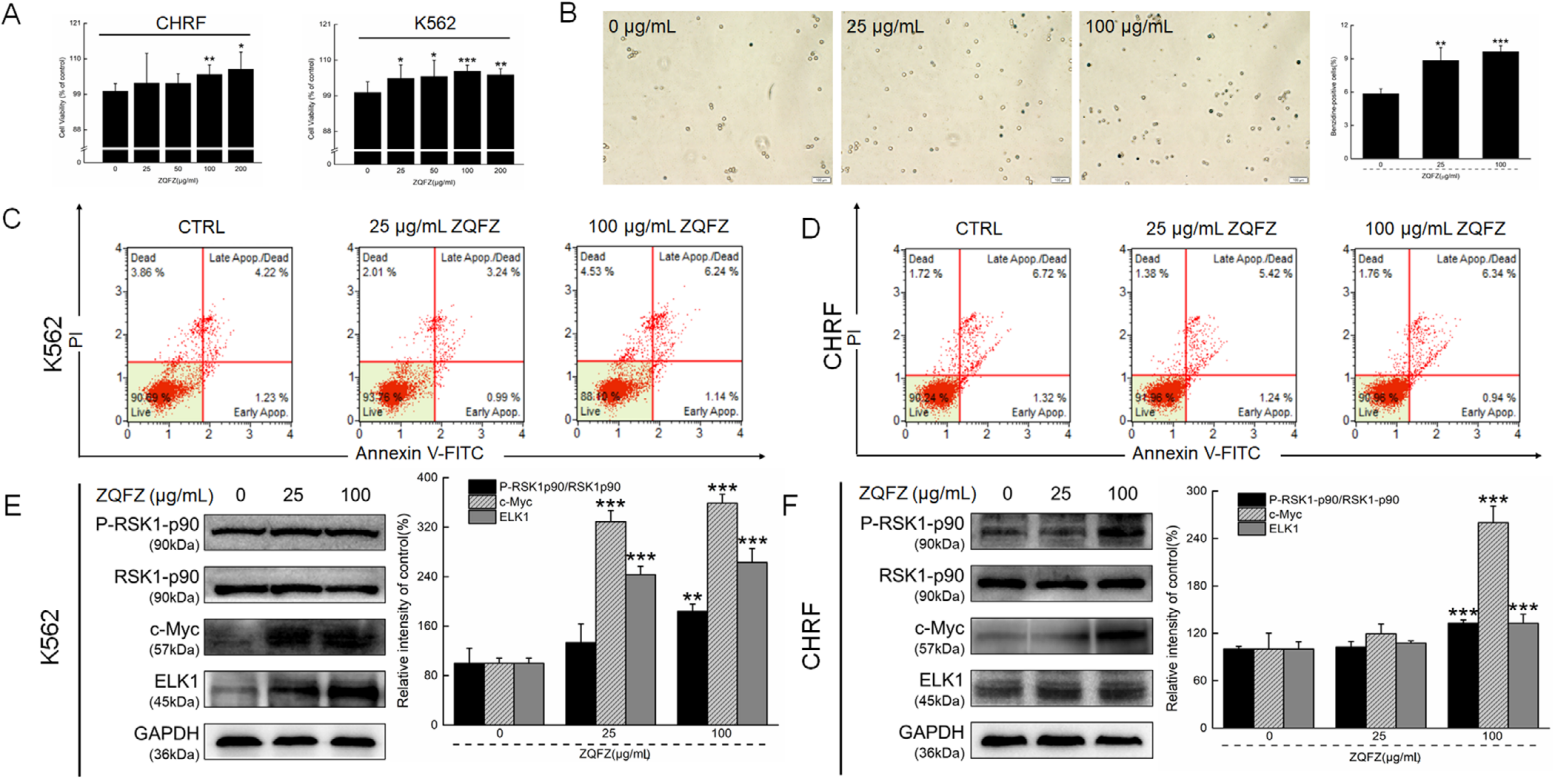
Potential properties of ZQFZ on the proliferation and differentiation of CHRF and/or K562 cells. The cells were incubated with ZQFZ at the dose of 0–200 μg/mL for 24 hours. (A) ZQFZ increased the proliferation of CHRF and K562 cells (n = 6). (B) ZQFZ motivated erythrocyte transformation of K562 cells analyzed by benzidine staining (10×; scale bar, 100 μm; n = 6). (C) ZQFZ showed no effects on the apoptotic rate of K562 and (D) CHRF cells detected by Annexin V/PI staining (n = 6). ZQFZ regulated the expression levels of P‐RSK1p90, c‐Myc, and ELK1 in (E) K562 cells and (F) CHRF cells detected by Western blotting (n = 6). The quantitative data of the expression levels of target proteins were normalized against the corresponding expression levels of GAPDH and the related total protein. Data are shown as the mean ± standard deviation (n = 6) and were analyzed by a one‐way analysis of variance followed by Tukey's test. *P < .05, ** *P* < .01, and *** *P* < .001 vs. 0 μg/mL ZQFZ‐treated cells. ZQFZ, ZhenQi FuZheng formula

**FIGURE 2:**
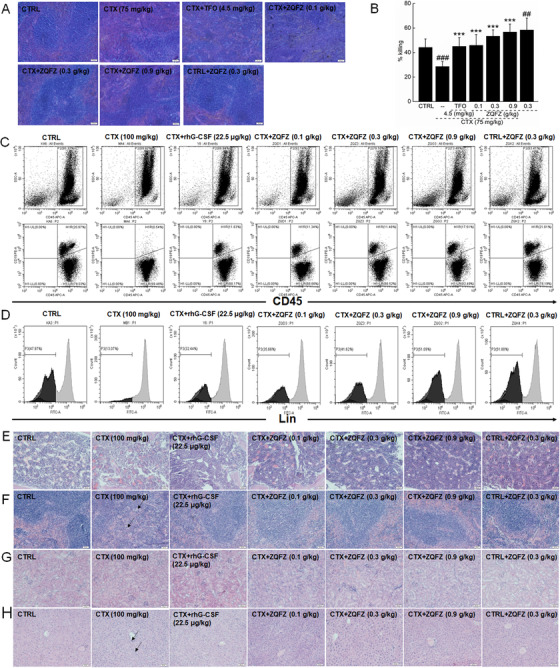
(A) H&E staining procedure was used to evaluate the pathological alterations in spleen of CTX‐injected mice with immunosuppression (20 ×, scale bar: 50 μm) (n = 5). (B) ZQFZ enhanced the activities of natural killer cells in CTX‐injected mice with immunosuppression. Data are shown as the mean ± SD (n = 10) and were analyzed by a one‐way analysis of variance followed by Tukey's test. ##*P* < .01 and ###*P* < .001 versus the control group, ***P < 0.001 versus the model group. (C) ZQFZ and rhG‐CSF improved the hematopoietic function in CTX‐injected mice. Flow cytometry was used to analyze the proportion of leukocytes in the murine bone marrow. CD45 was used to sort leukocytes. CD45^+^ CD19^+^ represents the B lymphocytes. (D) Percentage of blasts (Lin^‐^) in the murine bone marrow of CTX‐injected mice was analyzed using a flow cytometry assay. H&E staining procedure was used to evaluate the pathological alterations in (E) cellularity of bone marrow, (F) spleen (arrows show the multinucleated giant cells), (G) kidneys, and (H) liver (arrows show the inflammatory infiltration phenomenon) under a lightmicroscope digital camera (20 ×, scale bar: 50 μm). CTX, cyclophosphamide; ZQFZ, ZhenQi FuZheng formula; rhG‐CSF, recombinant human granulocyte colony‐stimulating factor

In immunosuppressed mice established using cyclophosphamide (CTX) (75 mg/kg) injection, ZQFZ influenced the levels of immune function‐related factors, including interleukins (ILs) and other serum cytokines (Table S1), relieved kidney damage (Figure 2A), and enhanced the activities of natural killer cells (Figure 2B), thereby demonstrating the immunoregulatory function of ZQFZ. The strong regulation of ILs, such as IL‐2, inspired us to conduct further research owing to their stimulatory effect on the growth of T and B cells.^2^

In hematopoiesis‐damaged mice established using CTX (100 mg/kg) injection, ZQFZ administration resulted in the enhancement of the proportions of CD19^+^, CD45^+^ (Figure 2C), and Lin^–^ (Figure 2D) cells in the bone marrow mononuclear cell fraction. CD45 can activate B lymphocytes owing to its synergistic effect, whereas CD19, which is mainly expressed on B lymphocytes, helps to regulate their maturation and differentiation.^3^ B lymphocytes, which mainly originate from HSCs, maintain the function of bone marrow.^4^ In the bone marrow of mice with hematopoietic failure, ZQFZ increased the proportion of cells and prevented their destruction (Figure 2E); meanwhile, ZQFZ improved the functions of the spleen (Figure 2F), kidneys (Figure 2G), and liver (Figure 2H). In the peripheral blood, ZQFZ restored the pathological alterations caused by CTX (Table S2). All of these findings support that ZQFZ improves hematopoietic function.

The differentiation and maturation of hematopoietic cells are mainly controlled by cytokines, including ILs and interferons.^5^ Host cells of the hematopoietic system can be injured by chronic chemotherapy/radiotherapy, leading to the release of proinflammatory factors, especially tumor necrosis factor (TNF‐α), which can directly suppress the function of the hematopoietic system by disturbing the activation of HSCs and reducing the proportion of bone marrow precursors.^4^ The detection of cytokine expression in the spleens and sera of mice with hematopoietic dysfunction using the Mouse Cytokine Array Kit and enzyme‐linked immunosorbent assay indicated that ZQFZ boosted the serum concentrations of macrophage colony‐stimulating factor (M‐CSF), and ILs and suppressed the serum concentration of TNF‐α in mice with hematopoietic failure (Figure 3A and B; Table S3). M‐CSF, a hematopoietic growth factor, is involved in the proliferation, differentiation, and survival of monocytes and macrophages, which not only control the levels of ILs and TNF‐α,^6^ but also regulate the expression of proteins such as ELK1, c‐Myc, and RSK by indirectly activating extracellular signal‐regulated kinase (ERKs).^7^ According to previous research, M‐CSF treatment can directly induce the myeloid master regulator PU.1 and instruct the myeloid cell‐fate change in HSCs independently of selective survival or proliferation in both in vitro and in vivo experiments.^8^ In M‐CSF‐siRNA‐transfected K562 cells, the enhancement of erythrocyte transformation (Figure 3D) and the expressions of P‐RSK1‐p90, c‐Myc, and ELK1 (Figure 3E) by ZQFZ were all strongly abolished. All of these findings suggest that the ZQFZ‐mediated improvement of hematopoietic function is at least partially related to the upregulation of M‐CSF levels.

**FIGURE 3:**
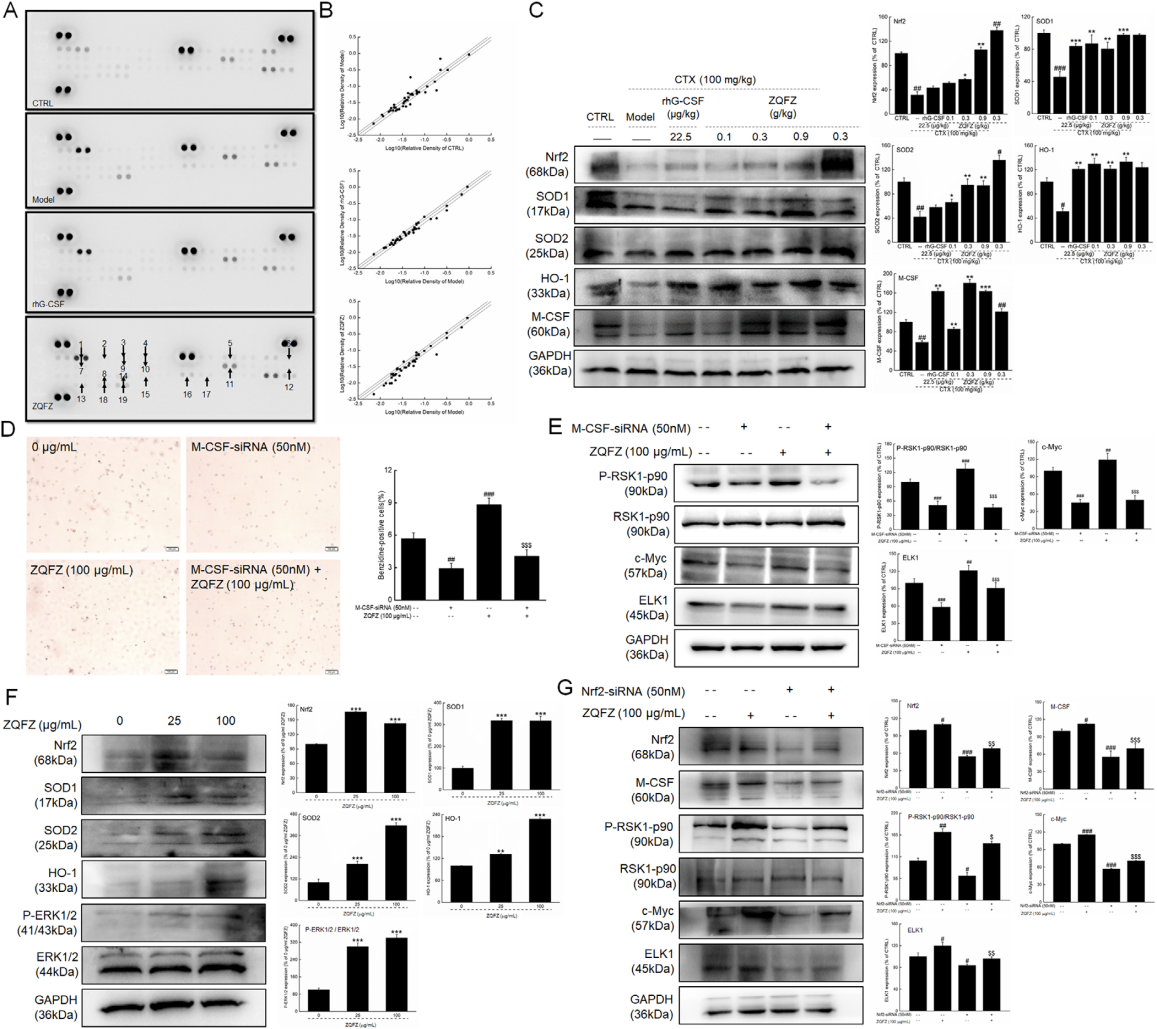
(A) Nrf2 plays important roles in the protective effect of ZQFZ against CTX‐induced hematopoietic dysfunction. Graphical representation of cytokine expression in the spleens of mice with hematopoietic dysfunction detected using the Mouse Cytokine Array Panel A Kit. Arrows indicate factors with a change of more than 20% (ZQFZ group vs model group). 1: C5/C5a; 2: G‐CSF; 3: GM‐CSF; 4: I‐309; 5: IL‐1α; 6: IL‐ 2; 7: IL‐4; 8: IL‐5; 9: IL‐6; 10: IL‐7; 11: IL‐16; 12: IL‐27; 13: I‐TAC; 14: M‐CSF; 15: JE; 16: MIG; 17: MIP‐ 1α; 18: TNF‐α; 19: TIMP‐1. (B) Scatter diagram of the 40 cytokines detected in the spleens of hematopoiesis‐damaged mice using the Mouse Cytokine Array Panel A Kit. The relative density is the ratio of the absolute value and the reference spot value. (C) ZQFZ regulated the expression levels of Nrf2, SOD1, SOD2, HO‐1, and M‐CSF in the spleens of CTX‐injected mice with hematopoietic dysfunction. (D) M‐CSF‐siRNA transfection strongly abolished the erythrocyte transformation of K562 cells caused by ZQFZ. (E) M‐CSF‐siRNA transfection strongly suppresses the expression of P‐RSK1p90, c‐Myc, and ELK1 in ZQFZ‐treated K562 cells. (F) ZQFZ changed the expression levels of target proteins in primary cultured bone marrow cells including P‐ERK1/2, Nrf2, SOD1, SOD2, and HO‐1. (G) The upregulation of the expressions of Nrf2, M‐CSF, P‐RSK1p90, c‐Myc, and ELK1 by ZQFZ was strongly abolished in Nrf2‐siRNA‐transfected K562 cells. The quantitative data of the expression levels of target proteins were normalized against the corresponding expression levels of GAPDH and the related total protein. Data are shown as the mean ± SD (n = 6) and were analyzed by a one‐way analysis of variance followed by Tukey's test. #*P* < .05, ##*P* < .01, and ### *P* < .001 versus control mice (for C) and control K562 cells (for D, E, and G); $*P* < .05, $$*P* < .01, and $$$ *P* < .001 versus ZQFZ‐treated K562 cells (for D, E, and G). *P < .05, **P < .01, and ***P < .001 versus model mice (for C) or 0 μg/mL ZQFZ‐treated primary cultured bone marrow cells (for F). CTX, cyclophosphamide; M‐CSF, macrophage colony‐stimulating factor; ZQFZ, ZhenQi FuZheng formula

During oxidative stress, the overproduction of reactive oxygen species (ROS) is toxic to HSCs and damages mature erythrocytes. In hematopoiesis‐damaged mice treated with ZQFZ, the splenic ROS levels were strongly suppressed (Table S3), which promoted the expressions of nuclear factor erythroid 2‐related factor 2 (Nrf2) (Figure 3C). Nrf2 can directly increase the levels of hemeoxygenase‐1, (SOD)‐1, and SOD‐2, all of which can suppress the ROS production as a feedback loop.^9^ The high expression of Nrf2 can promote the serum concentration of M‐CSF.^10^ In primary cultured bone marrow cells, ZQFZ upregulated the expression of Nrf2 and its downstream proteins, thereby further confirming the important role of Nrf2 signaling (Figure 3F). We further found that in Nrf2‐siRNA‐transfected K562 cells, the enhanced levels of M‐CSF and erythroid differentiation‐related proteins after ZQFZ exposure were all abolished (Figure 3G). This indicated that the ZQFZ‐mediated improvement of hematopoietic function via M‐CSF might have been related to its modulatory effect of Nrf2 activation.

Several limitations need to be further investigated. As a Chinese medicinal formula, ZQFZ contains multiple functional ingredients, some of which have been detected by HPLC. Owing to the properties of its crude ingredients, its effects were found to occur in a nondose‐dependent manner. Although we confirmed the auxo‐action of ZQFZ on immunoregulation and hematopoietic function, we failed to reveal their linkage.

In conclusion, this study confirmed the improvement of the hematopoietic system by ZQFZ in CTX‐injected mice with immunosuppression and hematopoietic dysfunction. These effects are related to the enhancement of the levels of M‐CSF by ZQFZ partially through the activation of Nrf2 signaling. All of our data support the application of ZQFZ not only as an immunomodulator, but also as an anti‐myelosuppressive agent.

## ETHICAL APPROVAL

The experimental protocol has been approved by the Institution Animal Ethics Committee of Jilin University (2017SY0603).

## Funding information

Special Projects of Cooperation between Jilin University and Jilin Province in China; Grant Numbers: SXGJSF2017‐1 and SXGJSFKT2020‐1; Science and Technology Develop Project in Jilin Province of China; Grant Numbers: 20191102027YY, 20200708091YY and 20200708068YY.

## AUTHOR CONTRIBUTIONS

Di Wang and Yaping Tian conceived and performed the experiments, and designed and revised the manuscript. Dongjie Li, Qiubo Chu, Shimiao Wang, Lanzhou Li, Bo Dou, and Jiawei He performed the experiments, contributed to the analysis of data, and writing of the manuscript.

## CONFLICT OF INTEREST

The authors declare that there is no conflict of interest.

## Supporting information

SUPPORTING INFORMATION

## Data Availability

All data generated and analyzed during the present study are included in this published article.
