# Supplementary material for: ZhenQi FuZheng formula‐mediated improvement of hematopoietic function in cyclophosphamide‐treated mice via the upregulation of macrophage colony‐stimulating factor concentrations
Source: Clin Transl Med. 2020 Dec 16;10(8):e256. doi: 10.1002/ctm2.256 (PMC7744025; doi:10.1002/ctm2.256)
Supplement: Supplementary file 1 — SUPPORTING INFORMATION [file CTM2-10-e256-s001.doc]

**Supplemental Material**

**ZhenQi FuZheng formula-mediated improvement of hematopoietic function in cyclophosphamide-treated mice via the upregulation of macrophage colony-stimulating factor concentrations**

Dongjie Li^1, 2#^, Qiubo Chu^2#^, Shimiao Wang^2#^, Lanzhou Li^2^, Bo Dou^2^, Jiawei He^2^, Yaping Tian^3^*, Di Wang^2^*

1. *Department of Otorhinolaryngology Head and Neck Surgery, the First Hospital of Jilin University, Changchun, Jilin 130021, P.R.China;*
2. *School of Life Sciences, Jilin University, Changchun, Jilin 130012, P.R.China;*
3. *Department of Dermatology and Venerology, the First Hospital of Jilin University, Changchun, Jilin 130021, P.R.China.*

Supplemental Figure S1………………………………………………………………2

Supplemental Table S1……………………………………………………………….3

Supplemental Table S2…………………………………………………………….…4

Supplemental Table S3……………………………………………………………….5


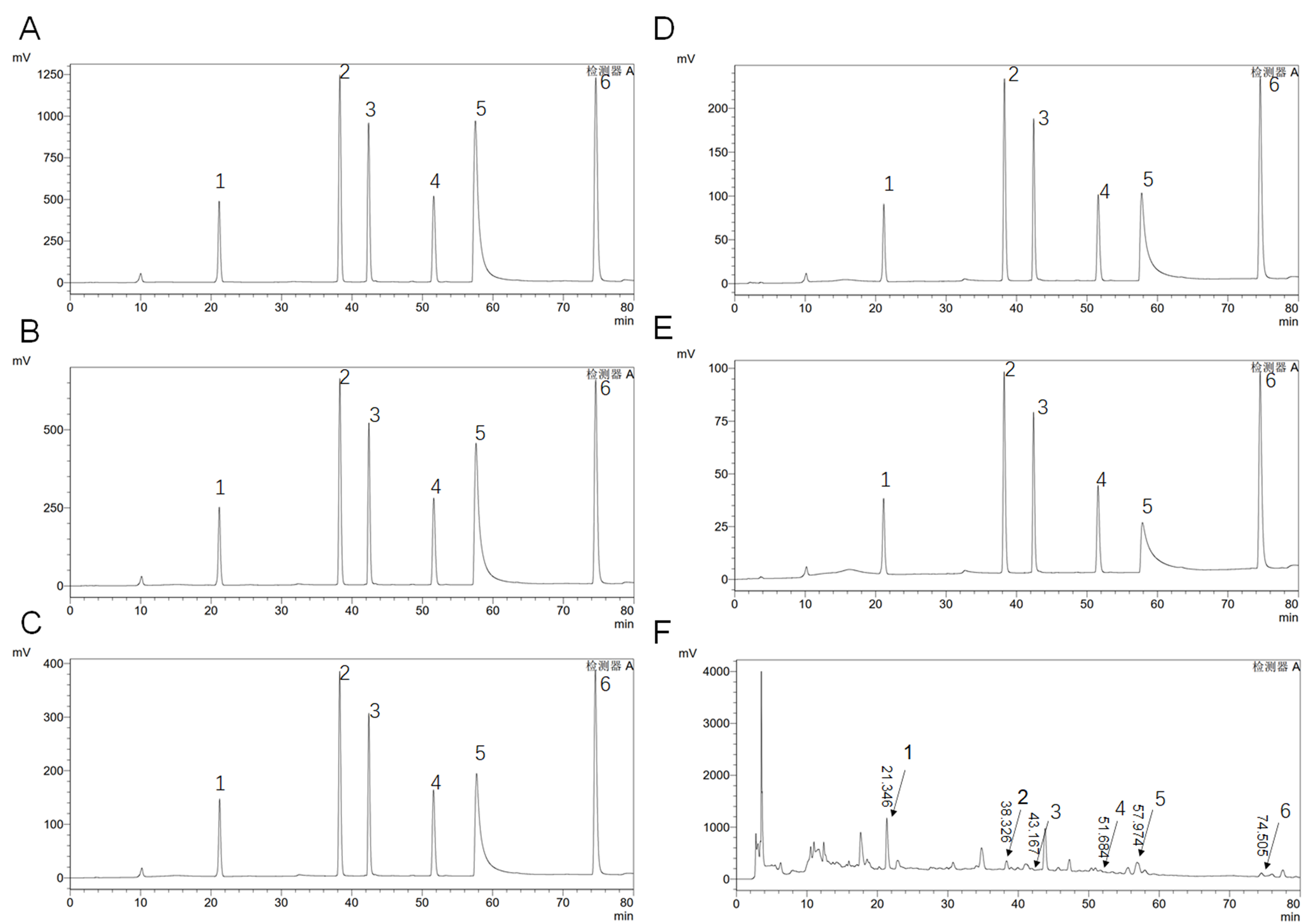


**Supplemental Figure S1.** Chromatograms of mixture standard and ZQFZ. The concentration of mixture standard from 1 to 6 is (**A**) 0.6, 0.6, 0.3, 0.25, 0.8 and 0.4 mg/ml, (**B**) 0.3, 0.3, 0.15, 0.125, 0.4 and 0.2 mg/ml, (**C**) 0.15, 0.15, 0.075, 0.0625, 0.2 and 0.1 mg/ml, (**D**) 0.075, 0.075, 0.0375, 0.03125, 0.1 and 0.05 mg/ml, and (**E**) 0.0375, 0.0375, 0.01875, 0.015625, 0.05 and 0.025 mg/ml. (**F**) The chromatogram of 0.5 g/ml of ZQFZ. 1. Salidroside, 2. Calycosin-7-glucoside, 3. Ligustroflavone, 4. Ononin, 5. Quercetin, 6. formononetin. ZQFZ, ZhenQi FuZheng formula.

**Supplemental Table S1.** The effects of ZQFZ on serum cytokines of CTX-injected mice with immunosuppression.

|  | CTRL | CTX (75 mg/kg) | | | | |  |
| --- | --- | --- | --- | --- | --- | --- | --- |
|  |  | -- | TFO (mg/kg) | ZQFZ (g/kg) | | | |
|  |  |  | 4.5 | 0.1 | 0.3 | 0.9 | 0.3 |
| IgA (μg/mL) | 92.0±8.6 | 83.9±8.0^#^ | 87.6±9.0 | 87.5±6.2 | 88.8±7.9 | 94.3±7.8^*^ | 90.4±15.9 |
| IgG (mg/mL) | 7.2±0.7 | 6.1±0.9^##^ | 6.4±0.9 | 7.0±0.8 | 7.4±0.9^**^ | 7.4±0.8^**^ | 7.4±0.6 |
| IgM (μg/mL) | 1005.4±70.8 | 863.7±38.4^###^ | 954.4±67.3^**^ | 843.8±65.0 | 861.9±61.2 | 965.6±80.8^*^ | 935.6±64.6 |
| IL-1β (pg/mL) | 31.2±3.0 | 34.0±2.0^#^ | 28.3±4.1^**^ | 32.8±3.5 | 28.8±6.2^*^ | 27.4±4.2^***^ | 33.5±1.3 |
| IL-2 (pg/mL) | 72.9±7.1 | 64.5±8.0^#^ | 74.3±6.2^**^ | 81.7±7.5^***^ | 83.2±7.2^***^ | 83.3±9.1^***^ | 81.9±10.4^#^ |
| IL-6 (pg/mL) | 40.2±3.7 | 33.3±2.9^###^ | 36.6±3.8^*^ | 37.3±2.4^**^ | 37.1±3.5^*^ | 36.6±2.0^*^ | 38.4±1.6 |
| IL-10 (pg/mL) | 358.0±15.9 | 335.2±16.9^#^ | 347.3±17.6 | 341.6±27.1 | 341.8±26.2 | 367.7±12.2^**^ | 347.2±12.6 |
| TNF-β (pg/mL) | 59.8±3.9 | 63.8±4.1^#^ | 58.1±5.3^*^ | 57.8±5.8^*^ | 60.5±7.7 | 63.5±8.8 | 58.1±15.6 |
| IFN-α (pg/mL) | 12.9±1.0 | 11.1±1.0^###^ | 11.8±0.8 | 11.7±0.7 | 11.9±1.3 | 13.2±1.9^**^ | 12.0±1.9 |

Data are expressed as mean ± S.D. (n = 10/group) and analyzed using a one-way analysis of variance followed by Tukey’s test. ^#^*P* < 0.05, ^##^*P* < 0.01 and ^###^*P* < 0.001 vs. control group, ^*^*P* < 0.05, ^**^*P* < 0.01 and ^***^*P* < 0.001 vs. model group. ZQFZ, ZhenQi FuZheng formula; CTX, cyclophosphamide; TFO, transfer factor oral liquid.

**Supplemental Table S2.** The effects of ZQFZ on peripheral blood cells of CTX-injected mice with hematopoietic dysfunction.

|  | CTRL | CTX (100 mg/kg) | | | | |  |
| --- | --- | --- | --- | --- | --- | --- | --- |
|  |  | -- | rhG-CSF (μg/kg) | ZQFZ (g/kg) | | | |
|  |  |  | 22.5 | 0.1 | 0.3 | 0.9 | 0.3 |
| NE (%) | 16.5±0.7 | 12.8±1.2^#^ | 25±9.0 | 40.7±2.6*** | 44.8±4.4*** | 37.1±2.0*** | 18.2±5.4 |
| LY (%) | 54.6±8.2 | 17.8±4.0^###^ | 16.5±4.0 | 19.0±7.6 | 29.8±1.5* | 32.2±6.0* | 51±6.7 |
| MO (%) | 26.9±4.0 | 65.7±4.8^###^ | 61.1±6.1 | 38.8±8.3** | 34.1±5.2*** | 30.2±5.7*** | 23.7±5.3 |
| HGB (g/L) | 148.3±6.8 | 127±3.6^##^ | 125±9.0 | 114.3±12.1 | 128±7.5 | 141.7±2.5** | 149±4.6 |
| MCV (fL) | 44.3±1.7 | 42.6±1.6 | 41.1±1.8 | 41.8±0.8 | 42.5±0.5 | 43.9±1.6 | 43±0.7 |
| MCH (pg) | 15.1±0.2 | 13.5±0.3^###^ | 13.2±0.7 | 13.7±0.3 | 14.4±0.3* | 15.6±0.4*** | 14.7±0.4 |
| MCHC (g/L) | 336.8±7.9 | 317±20.1 | 321.5±5.1 | 327.7±3.5 | 338.3±4 | 356±3.6* | 341.7±12.3 |

Data are showed as the means ± S.D. (n = 10/group) and analyzed using a one-way analysis of variance followed by Tukey’s test. ^#^*P* < 0.05, ^##^*P* < 0.01 and ^###^*P* < 0.001 vs. control group, ^*^*P* < 0.05, ^**^*P* < 0.01 and ^***^*P* < 0.001 vs. model group. ZQFZ, ZhenQi FuZheng fomula; CTX, cyclophosphamide; rhG-CSF, recombinant human granulocyte colony-stimulating factor; NE, Norepinephrine; LY, lymphocyte; MO, monocytes; HGB, hemoglobin; MCV, erythrocyte mean corpuscular volume; MCH, mean corpuscular hemoglobin; MCHC, mean corpuscular hemoglobin concentration.

|  |  | CTRL | CTX (100 mg/kg) | | | | |  |
| --- | --- | --- | --- | --- | --- | --- | --- | --- |
|  |  |  | -- | rhG-CSF (μg/kg) | ZQFZ (g/kg) | | | |
|  |  |  |  | 22.5 | 0.1 | 0.3 | 0.9 | 0.3 |
| spleen | IL-2 (pg/mg) | 218.9±18.0 | 190.3±38.6^#^ | 228.8±35.0^*^ | 287.2±47.8^***^ | 286.5±23.7^***^ | 257.6±42.3^**^ | 258.7±35.0^##^ |
|  | IL-5 (pg/mg) | 4.9±0.4 | 3.4±1.3^##^ | 5.6±0.8^***^ | 6.2±0.4^***^ | 5.7±0.7^**^ | 5.6±0.6^**^ | 7.0±0.8^###^ |
|  | TNF-α (pg/mg) | 127.3±12.2 | 146.0±15.3^##^ | 132.4±15.3^*^ | 137.9±14.9 | 134.7±9.6 | 126.4±9.1^**^ | 138.8±15.2 |
|  | M-CSF (pg/mg) | 38.6±6.6 | 37.2±4.0 | 48.1±5.3^***^ | 55.7±6.7^***^ | 44.1±4.4^**^ | 51.0±4.6^***^ | 45.5±5.5 |
|  | ROS (μg/mg) | 60.9±6.9 | 87.4±10.4^###^ | 61.2±7.7^***^ | 71.5±15.5^*^ | 62.7±5.9^***^ | 54.9±7.2^***^ | 60.2±7.8 |
| serum | IL-2 (pg/mL) | 439.4±46.4 | 279.2±113.0^##^ | 293.5±45.8 | 446.3±77.6^**^ | 459.2±60.8^**^ | 479.7±112.3^**^ | 403.7±71.0 |
|  | IL-5 (ng/L) | 13.6±1.4 | 10.0±2.7^##^ | 15.4±2.5^**^ | 10.7±1.1 | 15.7±1.1^**^ | 14.5±1.9^**^ | 16.6±1.9^##^ |
|  | TNF-α (ng/L) | 609.9±65.2 | 677.6±46.2^##^ | 654.4±36.4 | 625.8±44.9^*^ | 627.6±44.6^*^ | 583.1±73.9^**^ | 575.2±57.2 |
|  | M-CSF (ng/L) | 49.5±7.6 | 44.3±5.2 | 60.1±14.1^**^ | 46.3±7.8 | 52.2±6.9^*^ | 51.4±4.3^**^ | 48.1±7.6 |

**Supplemental Table S3.** The effects of ZQFZ on cytokines of CTX-injected mice with hematopoietic dysfunction.

Data are expressed as mean ± S.D. (n = 10/group) and analyzed using a one-way analysis of variance followed by Tukey’s test. ^#^*P* < 0.05, ^##^*P* < 0.01 and ^###^*P* < 0.001 vs. control group, ^*^*P* < 0.05, ^**^*P* < 0.01 and ^***^*P* < 0.001 vs. model group. ZQFZ, ZhenQi FuZheng fomula; CTX, cyclophosphamide; rhG-CSF, recombinant human granulocyte colony-stimulating factor.
